# Evolution of Human Brain Atlases in Terms of Content, Applications, Functionality, and Availability

**DOI:** 10.1007/s12021-020-09481-9

**Published:** 2020-07-29

**Authors:** Wieslaw L. Nowinski

**Affiliations:** John Paul II Center for Virtual Anatomy and Surgical Simulation, University of Cardinal Stefan Wyszynski, Woycickiego 1/3, Block 12, room 1220, 01-938 Warsaw, Poland

**Keywords:** Human brain atlas, Brain atlas evolution, Brain atlas generations, Brain atlas review, Brain atlas platforms

## Abstract

Human brain atlases have been evolving tremendously, propelled recently by brain big projects, and driven by sophisticated imaging techniques, advanced brain mapping methods, vast data, analytical strategies, and powerful computing. We overview here this evolution in four categories: content, applications, functionality, and availability, in contrast to other works limited mostly to content. Four atlas generations are distinguished: early cortical maps, print stereotactic atlases, early digital atlases, and advanced brain atlas platforms, and 5 avenues in electronic atlases spanning the last two generations. Content-wise, new electronic atlases are categorized into eight groups considering their scope, parcellation, modality, plurality, scale, ethnicity, abnormality, and a mixture of them. Atlas content developments in these groups are heading in 23 various directions. Application-wise, we overview atlases in neuroeducation, research, and clinics, including stereotactic and functional neurosurgery, neuroradiology, neurology, and stroke. Functionality-wise, tools and functionalities are addressed for atlas creation, navigation, individualization, enabling operations, and application-specific. Availability is discussed in media and platforms, ranging from mobile solutions to leading-edge supercomputers, with three accessibility levels. The major application-wise shift has been from research to clinical practice, particularly in stereotactic and functional neurosurgery, although clinical applications are still lagging behind the atlas content progress. Atlas functionality also has been relatively neglected until recently, as the management of brain data explosion requires powerful tools. We suggest that the future human brain atlas-related research and development activities shall be founded on and benefit from a standard framework containing the core virtual brain model cum the brain atlas platform general architecture.

## Introduction

We witness in recent years a tremendous explosion of human brain atlas projects with various goals, scopes, and sizes, as addressed, for instance, in (Amunts et al. 2014; Frackowiak and Markram 2015; Nowinski 2017a; Hess et al. 2018). This explosion is propelled by brain-related big and well-funded initiatives and projects, including *The BRAIN Initiative* (*Brain Research through Advancing Innovate Neurotechnologies*) (BRAIN Working Group 2014) to develop technology to catalyze neuroscience discovery (Jorgenson et al. 2015); *The Human Brain Project* to create a research infrastructure to decode the human brain, reconstruct the brain’s multiscale organization, and create brain-inspired information technology (Amunts et al. 2016); *The Human Connectome Project* to map structural and functional connections in the brain in order to study the relationship between brain circuits and behavior (Van Essen et al. 2013); *The Allen Brain Atlas* to map gene expression (Sunkin et al. 2013); *The Big Brain* to obtain ultra-high resolution neuroimages (Amunts et al. 2013); *The Blue Brain Project* to simulate neocortical micro-circuitry (Markram et al. 2015); *The CONNECT* project to combine macro- and micro-structure (Assaf et al. 2013); the *Brainnetome* project to understand the brain and its disorders, develop methods of brain network analysis at different scales, and create the brainnetome atlas (Jiang 2013); the *Chinese Color Nest Project* to study human connectomics across the life span (Zuo et al. 2017); and the Japanese *Brain/MINDS (Brain Mapping by Integrating Neurotechnologies for Disease Studies*) project to further understand the human brain and neuropsychiatric disorders through ‘‘translatable’’ biomarkers (Sadato et al. 2019). Therefore, with the new acquisition techniques introduced and big data acquired, sophisticated applications and tools developed, and novel concepts proposed, this explosion dynamically changes over time the concept, role, and understanding of a human brain atlas. Consequently, it is believed that the use of big digital science to neuroscience will create new avenues for the development of a modern human cerebral cartography (Frackowiak and Markram 2015). Two major forces driving this brain atlas development these are human curiosity along with scientific-based interest empowered by the developments in brain mapping technology and computing, and clinical needs urged by the growing cost of brain disorders and society aging.

The purpose of this state-of-the-art review is to attempt capturing the evolution of human brain atlases as well as to demonstrate the immense breadth of the ongoing work and its tremendous potential. We track this process of evolution over time, identify its numerous directions and categorize them, try to distinguish brain atlas generations, and capture the present state. Typically this evolution is considered in terms of atlas content, particularly, in research applications. However, the usefulness of human brain atlases depends not only on the atlas content but also on the functionality enabling and supporting various atlas-based applications as well as atlas availability. Therefore, our goal here is to take a wider perspective and address the human brain atlas development in terms of four major categories: (1) atlas content; (2) atlas applications in various areas; (3) functionality enabling and facilitating atlas use; and (4) availability in terms of access, media, and platforms enabling the atlas delivery to its user.

The rest of the paper is organized as follows. The brain atlas evolution is reviewed in Sect. 2 in terms of atlas content (categorized into 8 main groups from 23 directions for the new electronic brain atlases taking into account diverse criteria), applications, functionality, and availability. In Sect. 3 four generations of brain atlases are distinguished and the process of atlas evolution is captured diagrammatically, followed in Sect. 4 by the discussion along with some suggested future directions.

## Evolution of Human Brain Atlases

We track below the evolution of human brain atlases in terms of content, applications, functionality, and availability.

### Evolution of Brain Atlas Content

The brain atlas content is the richest and most dynamic category whose development proceeds along multiple divisions, including postmortem versus in vivo data, whole brain versus specific cerebral regions, structure versus function, single data acquisition modality versus multi-modal data, single brain specimen and individual features versus a population of specimens and/or aspects, in health versus diseased, static print versus dynamic digital, single atlas versus multi-atlases, slow versus fast dynamic, and mono scale versus multi-scale, among others.

The initial development of cerebral cortical maps was carried out predominantly in a single direction, meaning studying the cortical parcellation. Several early maps of the parcellated cerebral cortex were created in the first three decades of the 20th century by Campbell (1905), Brodmann (1909), Vogt and Vogt (1919), Flechsig (1920), and Von Economo and Koskinas (1925). These first, postmortem, hand-drawn cortical maps were produced for a single modality, cytoarchitectonics (Brodmann 1909; Von Economo and Koskinas 1925) or myeloarchitectonics (Vogt and Vogt 1919; Flechsig 1920), and they varied in terms of the number of the parcellated cortical areas. Namely, in the neocortex Campbell (1905) identified 14 areas, Brodmann (1909) 44 areas, Von Economo and Koskinas (1925) 54 areas, and Vogt and Vogt (1919) 185 areas. This process of cortical parcellation pioneered by Brodmann and the other early brain mappers a century ago continues until the present time being extended (1) from schematic two-dimensional (2D) single brain-derived surface drawings to multi-modal, population-based probabilistic three-dimensional (3D) maps (Glasser et al. 2016) facilitating to study intersubject variability; and (2) from pure visual inspection of the examined material to the application of robust, objective and observer-independent cortical parcellation rules based on quantitative criteria and statistical measures (Amunts and Zilles 2015), additionally enhanced by employing in vivo mapping with high-field magnetic resonance imaging (Geyer et al. 2011).

The need in neurosurgery to localize cerebral structures in the pre-tomographic imaging era caused the creation of stereotactic brain atlases (a review of print and electronic stereotactic atlases of the human brain is presented by Alho et al., (2011)). These initially print atlases represented a big step forward in atlas development both in terms of atlas content and concept. In the 1950th stereotactic brain atlases were produced by Speigel and Wycis (1952), Talairach et al. (1957), and Schaltenbrand and Bailey (1959). This development was continued by Andrew and Watkins (1969), Van Buren and Borke (1972), Schaltenbrand and Wahren (1977), Afshar et al. (1978), and Talairach and Tournoux (1988, 1993).

The major content-wise progress was made in four main directions: (1) from a few maps capturing the state-of-the-art about the brain to brain atlases applicable clinically; (2) from cerebral cortical maps to atlases of the whole brain (or its specific parts, including subcortical structures, cerebellum, and brainstem); (3) from a single specimen to multiple specimens with marked anatomic variability, although without any probabilistic maps yet (e.g., Schaltenbrand and Wahren (1977) used 111 brain specimens to create their atlas); and (4) integrating structure with function (neuroelectrophysiologic stimulation) like in the Schaltenbrand and Wahren atlas.

Besides stereotactic, some other print atlases were published for neuroradiology, neurosurgery, neuroscience, and medical education and training, among others, a brain atlas for computed tomography (Takayoshi and Hirano 1978), an atlas of the hippocampus (Duvernoy 1988), an atlas of brain function (Orrison 1995), an atlas of the brain stem and cerebellum with surface anatomy and vascularization (Duvernoy 1995), an atlas of morphology and functional neuroanatomy (Scarabino et al. 2006), an atlas of the brain stem and cerebellum with 9.4T images of 40–60 micron resolution (Naidich et al. 2009), and the Netter’s atlas of neuroscience (Felten et al, 2015). In particular, some stereo brain atlases are created as a 3D depth perception is essential in neurosurgical routine. Bassett (1952) produced a stereoscopic atlas of human anatomy with stereo cadaveric images of the central nervous system, head, and neck. Poletti (1985) built a stereo atlas of operative microneurosurgery with stereo photographs taken intraoperatively. Kraus and Bailey (1994) created a stereo atlas of microsurgical neuroanatomy with successive surgical steps recorded photographically; moreover, a binocular viewer is attached to the atlas to perceive depth.

A natural step forward in brain atlasing was the development of computerized electronic brain atlases aiming to overcome limitations of their print counterparts, such as static content, image plate sparseness, lack or limited functionality, cumbersome use, lack of interactivity, and difficulty in the mapping of the atlas content into an individual brain scan. These efforts have been headed at least in five directions: (1) direct digitization of the existing print atlases (Kall et al. 1985); (2) creation of bi-media atlases with both print and digital content (Zhang et al. 2003; Mai et al. 2004; Morel 2007); (3) 3D extension of the existing print atlases (Yoshida 1987; St-Jean et al. 1998); (4) creation of improved atlases derived from the print content by postprocessing, enhancements, and extensions (Nowinski et al. 1997a; Sudhyadhom et al. 2012); and (5) development of new electronic atlases (such as early ones, e.g., by Bohm et al. (1983) and Greitz et al. (1991) constructed from digitized crysection photographs, and many more created recently, as reviewed below). Note that the first two directions require no change in the original atlas content.

To our best knowledge the first computer program with digitized (and scalable) stereotactic atlases was developed by Bertrand et al. (1974). A digital version of the Schaltenbrand and Wahren atlas resident in a computer was created by Kall et al. (1985). Several groups developed electronic versions of the Schaltenbrand and Bailey atlas, namely, Yoshida (1987) built a 3D atlas by interpolating print plates, a 3D volumetric model of subcortical structures was produced by Kazarnovskaya et al. (1991), and Sudhyadhom et al. (2012) created a deformable 3D atlas for deep brain stimulation surgery by employing smoothing to reduce artifacts inherent in the print version. Similarly, digital versions of the Schaltenbrand and Wahren atlas were built and incorporated into atlas-aided software systems for stereotactic and functional neurosurgery by Sramka et al. (1997) and St-Jean et al. (1998), who developed a deformable volumetric version of the atlas.

We created a multi-brain atlas database with about 1000 structures and 400 sulcal patterns embedded into a neuroimaging system (Nowinski et al. 1997a) based on the content of four complementary classic Thieme brain atlases: Schaltenbrand and Wahren, Talairach and Tournoux (1988), Talairach and Tournoux (1993), and Ono et al. (1990)^1^. The original atlases were highly processed, manually edited, enhanced, fully segmented and labeled, extended including into 3D, and mutually spatially co-registered. Various content representations were created, including color-coded, contour (closed for structures and open for sulcal patterns), and polygonal. They facilitate atlas use and navigation and, particularly, the unique (color-coded or contour) representation enables automated labeling. A high-quality content along with a proposed method of atlas use (Nowinski 1998) caused the integration of this multi-brain atlas database into a majority of surgical workstations for clinical use (Nowinski 2009).

In addition, new dedicated neurosurgical atlases have been developed in the second decade of this century and they are discussed below.

Neuroeducation has driven the extension of the brain atlas content from 2D to 3D. The three-dimensional effect has been achieved by various techniques ranging from a simple form of virtual reality (VR) through QuickTime VR technology (Kling-Petersen and Rydmark 1997) to visualization of truly 3D representations by employing volume rendering of volumetric data (Hoehne et al. 1992) and surface rendering of geometric (polygonal) models (Nowinski et al. 2011b). The latter approach provides fast rendering of geometric models created with subpixel resolution, e.g., as small as 1/10th of the pixel size (Nowinski et al. 2012a). An overview of methods for 3D visualization of neuroanatomical image data and reconstruction of neuronal structures in brain atlases is presented by Maye et al. (2006).

Atlas-assisted neuroeducation, training, and simulation have greatly benefitted from the Visible Human Project (VHP) comprising the most complete volumetric data of human anatomy, including cryosection photographs, computed tomography and magnetic resonance images of American male and female specimens (Spitzer et al. 1996). The VHP provides excellent source material for the creation of brain atlases and maps, for instance by Drury and Van Essen (1997) and Juanes et al. (2012). The VHP additionally sparked other projects, including Chinese VHP and Korean VHP, resulting in the construction of new atlases (Zhang et al. 2003; Li et al. 2014) along with suitable tools for sectional and surface anatomy navigation as well as virtual dissection and endoscopy simulation (Chung and Park 2007).

Tremendous advancements in imaging, brain mapping, and computing propelled the development of new human electronic brain atlases. Various criteria can be employed to identify and systemize multiple directions in the content evolution of new atlases, including parcellation, modality, plurality, quality, ab/normality, lifespan, extendibility, ethnicity, spatial and temporal scales, integration, transformation, techniques of creation, and combination of them. We determine 23 directions and categorize them into eight (seven main and one combined) groups of brain atlas content development. Then, by taking into account this categorization, a brain atlas instant can be considered as an element in a seven-dimensional brain atlas space. These groups along with their component directions are as follows:

- Scope (content extent)
- *From cerebral parts* (e.g., the basal ganglia (Yelnik et al. 2007), thalamus and basal ganglia (Morel 2007), thalamus (Krauth et al. 2010), and deep brain structures (Lemaire et al. 2019)) *to the whole brain* (Kikinis et al. 1996; Hoehne 2001; Tzourio-Mazoyer et al, 2002; Nowinski et al. 2011b);
- *From structural neuroanatomy* (Rohlfing et al. 2010; Mandal et al. 2012; Nowinski and Chua 2014) *to vascular neuroanatomy* (Nowinski et al. 2009b; 2011a; Huck et al. 2019) *to connectional neuroanatomy* (Mori et al. 2005; Nowinski et al. 2012b; Van Essen 2013; Van Essen et al. 2013; Baker et al. 2018; Briggs et al. 2018) *to gene expression* (Sunkin et al. 2013) including gene expression in brain development (Kanton et al. 2019);
- *From brain to head* (Tiede et al. 1996; Chen et al. 2018), *and to head and neck* (Nowinski 2017b);
- *From structure to function*, including functional atlases (Minoshima et al. 1994; Zhao et al. 2017; Haegelen et al. 2018; Varoquaux et al. 2018; Lehman et al. 2020), integrated anatomic-functional atlases (Nowinski 2004; Nowinski et al. 2010), and functional connectivity atlases (Craddock et al. 2012; James et al. 2016).
- Parcellation
  *Use of diverse, often multiple parcellation criteria*, from classic cytoarchitecture, myeloarchitecture and gross anatomy to fMRI, chemoarchitecture (Yelnik et al. 2007), vascular territories (Nowinski et al. 2006), anatomic connectivity (Mori et al. 2005), functional connectivity (Arsiwalla et al. 2015), anatomic-functional connectivity (Fan et al. 2016), (multi)receptor architecture (Amunts et al. 2010), and/or multiplicity of them (Van Essen 2013; Glasser et al. 2016), among others.
- Modality
- *From postmortem to in vivo data* (Lehmann et al. 1991; Nowinski et al. 2015a; Dickie et al. 2017; Oishi et al. 2019);
- *Integrating postmortem – in vivo data* (Nowinski et al. 1997b; 2002b; Yelnik et al. 2007; Cho et al. 2008; Amunts et al. 2014);
- *Increased teslage*, from 1.5T (Tesla) (Hoehne 2001) to 3T (Nowinski et al. 2009b; Rohlfing et al. 2010) to 7T (Cho et al. 2008; Nowinski et al. 2015a; Saygin et al. 2017; Huck et al. 2019; Liu et al. 2020) to 9.4T (Yushkevich et al. 2009);
- *From image to non-image data*, transforming into brain atlases non-image data, such as stimulating electrode geometry (Nowinski et al. 2003) and neurologic parameters (Nowinski et al. 2014a).
- Plurality
- *Specimen-related*: from a single specimen to population atlases for cerebral parts (such as the cerebellar nuclei (Dimitrova et al. 2006), insula (Faillenot et al. 2017), cortical structures (Shattuck et al. 2008), and cerebral arteries (Dunås et al. 2017) to the whole human brain (Mazziotta et al. 1995; 2001; Thompson et al. 2000);
- *Variant-related*: from a single variant to a collection of variants, for instance, the cerebrovascular variants (Nowinski et al. 2009a);
- *Modality-related*: from uni-modal to multi-modal atlases with the use of multi-modal complementary data (e.g., Johnson and Becker 1999; Toga et al. 2006; Nowinski et al. 2010; Hawrylycz et al. 2012; Ding et al. 2016);
- *Channel*-related: e.g., with anatomy, diffusion, and tissue channels (Rohlfing et al. 2010);
- *Atlas-related*: from a single atlas to arrays of fully parcellated atlases or mega multi-atlases (Wu et al. 2016).
- Scale
- *Spatial scale*, *from macro- to meso- to micro- to nano-scales* along with integrating atlas data across multiple spatial scales (Assaf et al. 2013; Ding et al. 2016; Ecker et al. 2017);
- *Temporal scale* covering atlases from development (Kanton et al. 2019) to lifespan including age-matched atlases to accommodate age-dependent anatomical changes ranging from pediatric to geriatric populations (Wu et al. 2016; Zuo et al. 2017; Zhang et al. 2018; Oishi et al. 2019);
- *Integrating spatio-temporal scales* (Sunkin et al. 2013; Bozek et al. 2018).
- Ethnicity
  Ethnic-specific atlases, for instance, for Chinese (Zhang et al. 2003), Korean (Cho et al. 2008), and Caucasian (Nowinski 2017b) specimens.
- Abnormality
  *From normal to disease-specific atlases* for various brain disorders, for instance, Alzheimer’s disease (Thompson et al. 2001), dementia (Mega et al. 2005), and stroke (Nowinski et al. 2014a; de Haan and Karnath 2017).
- Multiple (combined) groups
- *Population* multi-modal atlases (Iglesias et al. 2018);
- *Population functional maps and atlases* (Nowinski et al. 2003; Nowinski 2009; Breshears et al. 2015);
- *Population spatio-temporal atlases*, for instance, of brain development (Kuklisova-Murgasova, et al. 2011);
- *Population ethnic atlases and templates*, for instance, Chinese brain atlas (Tang et al. 2010), Indian brain template (Bhalerao et al. 2018) and atlas (Sivaswamy et al. 2019), Korean brain template (Lee et al. 2005), and French brain template (Lalys et al. 2010).

We witness recently an enormous development of population-based brain atlases both in health and disease. Population-based structural atlases have been built for the whole brain (Liang et al. 2015; Wu et al. 2016) and its specific regions, such as the cortical areas (Shattuck et al. 2008; Glasser et al. 2016), cerebellum (Diedrichsen et al. 2009), brainstem (Meola et al. 2016), subcortical nuclei (Pauli et al. 2018), thalamic nuclei (Iglesias et al. 2018; Najdenovska et al. 2018), insula (Faillenot et al. 2017), some gyri including the parietal lobe gyri (Wild et al. 2017) and the inferior frontal gyrus (Hammers et al. 2007), and venous cerebrovasculature (Huck et al. 2019).

More advanced atlases have been developed in terms of population (Liang et al. 2015), specimen age range span (Wu et al. 2016; Zhang et al. 2018), and age appropriateness (Fonov et al. 2011). For instance, the atlas of Chinese adults contains a large number of 2020 specimens whose age spans from 20 to 75 years at a 5-year interval (Liang et al. 2015). The longitudinal atlas for normative brain development and aging spans the age range of 1–83 years, while the quantitative susceptibility mapping used for its creation may facilitate the estimation of age-related iron changes in deep gray matter nuclei and myelin changes in white matter (Zhang et al. 2018). A mega multi-atlas (Wu et al. 2016) constitutes an inventory of 90 brain atlases with the specimens ranging from 4 to 82 years of age. Several age-dependent brain atlases have been built also for children (Ou et al. 2017; Bozek et al. 2018) and fetuses, such as a dynamic 4D (four-dimensional) probabilistic atlas of the developing brain (Kuklisova-Murgasova, et al. 2011). The baby brain atlases developed for specimens younger than 12 months old (for the fetus, neonate, and infant) are reviewed by Oishi et al. (2019).

Besides probabilistic structural atlases also a variety of probabilistic connectional atlases (Meola et al. 2016; Figley et al. 2017; Yeh et al. 2018; Chenot et al. 2019), functional maps and atlases (Nowinski et al. 2003; Nowinski 2009; Breshears et al. 2015), and vascular atlases (Dunås et al. 2017; Bernier et al. 2018; Mouches and Forkert 2019) have been created.

Developments in mapping the microscopical organization of the brain along with the progress in nanoscience (Alivisatos et al. 2013) enable the construction of brain maps and atlases across spatial scales extending from macro to meso to micro to nano. Examples include the *BigBrain* with 20-micrometer resolution (Amunts et al. 2013), a comprehensive cellular-resolution (of 1 µm/pixel) brain atlas linking macroscopic anatomical and microscopic cytoarchitectural parcellations (Ding et al. 2016), the *Brain Activity Map* as the functional connectome to elucidate emergent levels of neural circuit function (Alivisatos et al. 2012), a temporal cell atlas of gene expression in brain development (Kanton et al. 2019), a genomics brain atlas (Sunkin et al. 2013), a proteomic brain atlas (McKetney et al. 2019), an atlas of serotonin (Beliveau et al. 2017), and an atlas of brain transcriptome (Hawrylycz et al. 2012). In particular, identifying the different brain cell types to determine their roles in health and disease is of great importance and it is established as one of the six goals of the BRAIN Initiative (BRAIN Working Group 2014). Toward achieving this goal a whole-brain cell atlas is under development by Ecker et al. (2017) that integrates molecular, anatomical, and physiological annotations of neuronal cell types for a comprehensive characterization of cell types, their distributions, and patterns of connectivity.

### Evolution of Brain Atlas Applications

The rationale of creating the early cortical maps, the result of human curiosity, was to represent the knowledge of new discoveries about the human brain. The brain knowledge capturing, aggregation, and representation by means of human brain atlases has been the first application of the atlases, and this central role remains until the present. Research has been the dominant application of human brain atlases (Roland and Zilles 1994) employed as tools for analysis of brain structure and function (Hess et al. 2018), means to integrate neuroscience research data from healthy and diseased brains to increase data sharing and re-using (Bjerke et al. 2018), and a potential tool suitable for image structurization through atlas-based image parcellation to utilize a vast amount of imaging information available in medical record systems, such as the PACS (picture archiving and communication system) (Mori et al. 2013). Moreover, disease-specific atlases, such as (Thompson et al. 2001; Mega et al. 2005; de Haan and Karnath 2017) facilitate quantification of brain structural deficits in epilepsy, depression, schizophrenia, Alzheimer’s disease, bipolar disorders, autism and others disorders as discussed by Toga and Thompson (2005).

Human brain atlases are also useful beyond research in medical education and clinical applications. Stereotactic and functional neurosurgery was the first, major clinical application of brain atlases. We observe that every two decades mark major progress in this field. The first print atlases were created in the 1950th, the first digitized brain atlas was developed in the 1970th (Bertrand et al. 1974), and the acceptance of our electronic brain atlases to clinical practice by the community (and 13 surgical companies) started in the 1990th (Nowinski 2009). Initially, a digital atlas was used off-line for referencing while being placed beside the displayed patient-specific scan. In this way, for instance, *The Electronic Clinical Brain Atlas* (Nowinski et al. 1997b) had been employed next to a surgical workstation to plan neurosurgery before our brain atlas database was directly integrated with surgical workstations, such as the *StealthStation* (Nowinski 2009). In the second decade of this century several novel, neurosurgery-dedicated atlases have been developed for electrode placement in deep brain stimulation (Sadikot et al. 2011; Dergachyova et al. 2018; Haegelen et al. 2018; Nowacki et al. 2018).

In the pre-tomographic era, stereotactic brain atlases were useful to localize deep stereotactic targets. The introduction of diagnostic imaging has not eliminated brain atlases but rather changed their role and function (Nowinski 2009). Namely firstly, a high atlas parcellation, typically greater than that of a scan, allows the individualized atlas to facilitate targeting. Secondly, extensive atlas features in combination with its ease of use and precision facilitate neurosurgery planning and provide intraoperative support, like those available in *The Cerefy Clinical Brain Atlas: Extended Edition with Surgery Planning and Intraoperative Support* (Nowinski et al. 2005a). Thirdly, several new dedicated brain atlases have been created that are derived from various modalities including histology (Chakravarty et al. 2006), electrophysiology (Finnis et al. 2003; Nowinski et al. 2003), and multi-modalities (Yelnik et al. 2007; Nowinski et al. 2010; Haegelen et al. 2018).

Other examples of atlas use in neurosurgery include a digital brain atlas for surgical planning (Kikinis et al. 1996), an Internet portal for stereotactic and functional neurosurgery shifting the paradigm in atlas building from manufacturer-centric (dependent) to neurosurgical community-centric (Nowinski et al. 2002a), and a practical 3D atlas for a preoperative white matter-specific planning of subcortical trajectories (Jennings et al. 2018).

Several neuroeducational atlases were created in the 1990th, the *Decade of the Brain*, including *BrainStorm* (Dev et al. 1992), *Digital Anatomist* (Sundsten et al. 1994), *A.D.A.M.* (A.D.A.M 1996), *Microvascular Atlas of the Head and Neck* (Bayer 1996), *The BRAIN project* (Kling-Petersen and Rydmark 1997), and *The Electronic Clinical Brain Atlas* (Nowinski et al. 1997b).

These initial efforts were followed by the development of more advanced atlases in terms of content and functionality, such as *Voxel-man* (Hoehne 2001), *The Cerefy Atlas of Brain Anatomy* (Nowinski et al. 2002b), Primal’s *Interactive Head & Neck* (Berkovitz et al. 2003), *The Cerefy Clinical Brain Atlas* (Nowinski and Thirunavuukarasuu 2004), *The Cerefy Atlas of Cerebral Vasculature* (Nowinski et al. 2009b), *The Human Brain in 1492 Pieces* (Nowinski et al. 2011b), *The Human Brain in 1969 Pieces: Structure, Vasculature, Tracts, Cranial Nerves, Systems, Head Muscles, and Glands* (Nowinski and Chua 2014), *The Human Brain, Head and Neck in 2953 Pieces* (Nowinski et al. 2015a), and the *Human Anatomy Atlas* (Visible Body n.d.). In addition, individualized atlases that parcellate and annotate brain scans are useful for the creation of teaching files of brain anatomy and function (Oishi et al. 2019).

The brain atlases also play a role in training and simulation, e.g., in neurosurgery (Serra et al. 1997) and radiotherapy (Roniotis et al. 2012).

Human brain mapping in research and clinical practice is another major area of brain atlas employment. Digital brain atlases are exploited here to provide the underlying neuroanatomy and to automatically label activation loci in functional images with cortical areas and stereotactic coordinates. Application examples include the *BrainMap* (Lancaster et al. 2000) and the *Brain Atlas for Functional Imaging* (Nowinski et al. 2000b). Both these tools employ digital Brodmann’s areas (derived from the Talairach and Tournoux (1988) brain atlas) that are hidden in the *BrainMap* while explicitly available and displayed to the user in the *Brain Atlas for Functional Imaging*. Brodmann’s areas, despite being one century old and originally limited to two views on the visible part of the cortical surface only, are still today applicable references in human brain mapping to register functional activations to the underlying anatomy (Amunts and Zilles 2015). ). Because of well-known limitations of the Talairach and Tournoux atlas (see, e.g., (Nowinski and Thirunavuukarasuu, 2009)) in order to improve labeling of functional foci a dedicated AAL (Automated Anatomical Labeling) atlas was developed from a T1-weighted scan with 45 anatomical volumes of interest in each hemisphere (Tzourio-Mazoyer et al, 2002).

Nuclear medicine, such as SPECT (single-photon emission computed tomography) and PET (positron emission tomography), produces images of relatively poor spatial resolution, which makes it difficult to relate the functional information contained there to the corresponding underlying neuroanatomy. In order to enhance the accuracy and consistency of the anatomic interpretation of PET functional brain images, Minoshima et al. (1994) constructed a PET stereotactic brain atlas from a high-resolution [18F]FDG (fluorodeoxyglucose) images of a normal volunteer. In addition, to assist in the interpretation of SPECT scans of the brain, a 3D neuroanatomical atlas was created from an MRI scan of a normal, healthy volunteer by Lehmann et al. (1991).

Human brain atlases have potential in stroke management for prediction, diagnosis, and treatment (Nowinski 2020). The atlases of anatomy and blood supply territories support decision making in thrombolysis and provide a quantitative assessment of the infarct and penumbra (Nowinski et al. 2006). These two atlases also facilitate rapid and automatic detection, localization, and classification of ischemic and hemorrhagic lesions in the emergency room (Nowinski 2020). The probabilistic stroke atlas, created by the integration of brain scans with textual neurologic parameters of previously managed stroke patients, enables prediction of stroke outcomes (Nowinski et al. 2014a).

We have also developed atlas-based applications in several other areas, including neuroradiology, neurology, psychology, psychiatry, and proposed new solutions in some niche applications, such as atlas-guided do-it-yourself neurosurgery suitable for patients (Nowinski 2009) and an atlas-enhanced operating room for the future (Benabid and Nowinski 2003).

In neurology, the *3D Atlas of Neurologic Disorders* (Nowinski et al. 2014b) facilitates the understanding of neurologic deficits resulting from brain damage. The atlas bridges neuroanatomy, neuroradiology, and neurology (Nowinski and Chua 2013a). It serves as an educational means for neurology students and residents as well as a reference for neurologists. This atlas is also a potentially useful tool for psychologists, and particularly neuropsychologists, to communicate with patients.

The *Cerefy Neuroradiology Atlas* (Nowinski and Belov 2003) available over the Internet contains a fully segmented and labeled anatomic brain atlas. It provides functions for a rapid atlas-to-scan registration, interactive structure labeling and annotating, and mensuration. To our best knowledge, this is the first online, publicly available atlas-based application for neuroradiology. In general, brain atlases have a still unexploited potential in neuroradiology. For instance, in (Nowinski 2016) nine various scenarios of atlas use in neuroradiology were discussed based on the earlier developed working prototypes ranging from image interpretation to reporting to dealing with data explosion and to communication (for both doctor-to-doctor and doctor-to-patient).

In psychiatry, we employed a brain atlas to automatically generate anatomic volumes of interest for subsequent analysis in a population of schizophrenic patients and controls to study the passivity phenomenon (Sim et al. 2009).

### Development of Atlas Functionality

The early bare brain maps and atlases had no or a very limited supporting functionality. Therefore, certain early print stereotactic brain atlases, besides providing standard anatomical indices (along with some accompanying textual description), were placed in a stereotactic coordinate system enabling localization and referencing. Moreover, certain atlases were equipped with transparent overlays with structure annotations over the brain plates to facilitate structure delineation and identification. Conceptually, this simple functionality already signaled the necessity of equipping brain atlases with suitable tools enabling their clinical applications. This necessity in the pre-digital era was clearly expressed by a popular practice performed in the operating room of generating resized (individualized) brain atlas plates by means of an overhead projector and drawing a planned stereotactic trajectory directly on the displayed projection.

The need of generating individualized atlases in neurosurgery was met in the first computer program with digitized stereotactic atlases developed by Bertrand et al. (1974) that provided 1D (one-dimensional) atlas scaling along the inter-commissural distance. This solution was followed by a 3D piece-wise linear (Nowinski et al. 2000a) and non-linear (Ganser et al. 2004) brain atlas warping. The requirements from stereotactic and functional neurosurgery have been an initial major driving force behind the brain atlas development, both in terms of content (as discussed above) and functionality. Specific atlas-related tools have been proposed for pre-operative planning, intra-operative support, and postoperative assessment (Nowinski 2001a).

Pre-operatively, the atlas facilitates the target and trajectory planning to avoid some critical structures (such as the optic tract), and provides the list of trajectory-intersected structures. In order to increase both the quality of planning and the surgeon’s confidence, multiple complementary atlases are employed (Nowinski et al. 2000a; 2010). In general, the atlas facilitates the planning of the access corridor to any target structure by determining all the structures encountered along the selected corridor and those neighboring it, allowing the neurosurgeon to assess various potential corridors in the process of decision making. Intra-operatively, the atlas provides the actual structure where the tip of the electrode is located, the list of structures already intersected by the electrode, distances to critical structures, and the surrounding anatomic and vascular context (Nowinski et al. 2010). Additionally, the probabilistic functional atlas makes the targeting more accurate by determining the best location within the whole target structure (Nowinski et al. 2003; 2005b). Post-operatively, the atlas facilitates to analyze the correctness of placement of a stimulating electrode or a permanent lesion.

The first (to our best knowledge) collaborative use and construction of a brain atlas over the Internet by the neurosurgical community was offered in the portal for stereotactic and functional neurosurgery supporting a probabilistic functional atlas (Nowinski et al. 2002a). This atlas is calculated from neuroelectrophysiologic and neuroimaging patient-specific data acquired during functional neurosurgical procedures (Nowinski et al. 2003). The portal supports the atlas with the functionality enabling data uploading to the central database or downloading locally in order to combine them with the neurosurgeon’s own data, followed by the calculation of the individualized probabilistic functional atlas and surgery planning. The atlas is displayed graphically in 2D, 3D, and as a probability distribution histogram along with the data tree (including patients, electrodes, contacts, and coordinates) in a text format.

The development of atlas functionality has also been driven by the needs of the human brain mapping community, mainly for the integration of structural and functional, and, generally, multi-modal images in the same stereotactic space as well as for atlas-assisted automatic labeling of activation loci in functional images with cortical areas and stereotactic coordinates. The *SPM* anatomy toolbox (Eickhoff et al. 2005) is an example of an image integration tool that enables the combination of probabilistic cytoarchitectonic maps and results of functional imaging studies. Another data integration tool is the *Neuroinformatics Platform* within the *Human Brain Project* (Bjerke et al. 2018). Examples of labeling tools are the *BrainMap* (Lancaster et al. 2000) and the *Brain Atlas for Functional Imaging* (Nowinski et al. 2000b). The *BrainMap* assigns a label of the cortical area closest to the examined activation locus. This process is blind to the user as the brain atlas employed is hidden from display. In the *Brain Atlas for Functional Imaging* the parcellated, labeled, and color-coded cortical areas are explicitly available and displayed to the user, who has full control over the process of activation loci labeling and is able to edit their positions if needed. This atlas processes functional images through a locus-driven analysis (Nowinski and Thirunavuukarasuu 2003). The activation loci in functional images are extracted automatically by thresholding with the option of interactive editing. Then, the atlas of anatomy extended with Brodmann’s areas is employed for labeling of the activation loci with the names of cerebral structures, Brodmann’s areas, and stereotactic coordinates. The activation loci are marked on the images with the superimposed atlas, and the list of all labeled loci along with their values on the anatomic and functional images is provided to the user.

Neuroinformatics tools and repositories also have been developed to store various and heterogeneous results of analyses. For instance, *NeuroVault.org* stores these results in a form of statistical maps, parcellations, and atlases (Gorgolewski et al. 2016); and *BALSA* is a database of the brain analysis library of spatial maps and atlases (Van Essen et al. 2017).

Construction of population atlases as well as atlas-assisted neuroimage processing and analysis in any application requires atlas-to-scan (or scan-to-atlas) registration. Pioneering work on atlas-to-scan elastic registration was done by Bajcsy et al. (1983) and Gee et al. (1993). Brain image registration algorithms are evaluated, for instance, by Klein et al. (2009) and Ou et al. (2014). Image registration is also employed for multi-modal atlas construction and atlas-guided segmentation of brain images. Automatic segmentation of brain images, in particular, is of great importance and it can be performed through atlas-to-scan registration, as the individualized atlas segments and labels the underlying neuroanatomy. The majority of methods are for the segmentation of structural neuroanatomy, though some approaches are developed to provide atlas-based processing of connectional neuroanatomy (Labra et al. 2017) and cerebrovascular anatomy (Passat et al. 2006; Dunås et al. 2016). As multi-atlases are more powerful than single atlases, numerous approaches have been developed for multi-atlas based brain segmentation (Aljabar et al. 2009; Artaechevarria et al. 2009; Lötjönen et al. 2010; Wu et al. 2016; Zaffino et al. 2018; Li et al. 2019).

In neuroeducation a typical atlas-related functionality includes labeling, searching, and atlas display and manipulation. Beyond typical operations, some atlases provide more sophisticated operations, such as advanced labeling with vessel diameters (Nowinski et al. 2011b) and pathology description (Nowinski et al. 2014b) as well as quantification, such as geometric measurements (Nowinski et al. 2015a).

The atlas also enables automatic testing suitable for both self-testing and classroom testing. A testing module for atlas-enabled evaluation of brain knowledge in neuroeducation was designed and incorporated into *The Cerefy Atlas of Brain Anatomy* (Nowinski et al.  2002b), and the corresponding method presented in (Nowinski et al. 2009c). The module allows the instructor to set the testing parameters first, such as the scope of the tested knowledge, scoring points, and the number of attempts. The items (structures) in the index are consecutively numbered forming a list. A random generator selects randomly items from the list while avoiding repetition. There are two types of queries “Where is?” and “What is?” to test location and naming of cerebral structures, respectively. When the name of the selected item is highlighted in the index, the student is tested against “Where is?” aiming to point to the selected structure in the atlas (image or model). When the selected structure is highlighted in the atlas, the student is tested against “What is?” aiming to indicate the name of this structure in the index. After all the structures have randomly been selected, the module provides the total score and the time spent to perform the test. Note that for the same scope of a tested brain knowledge the queries, which are randomly generated, are different each time avoiding this way the situation that the student copies someone else’s answers or memorizes them.

Some other examples of application-specific functionality in our atlas-based solutions include brain scan interpretation in neuroradiology (Nowinski and Belov 2003), segmentation and labeling of pathological neuroimages (Nowinski and Belov 2005), automatic generation of atlas-derived regions and volumes of interest (VOI/ROI) for fast comparison of the left and right cerebral hemispheres to detect pathology (Nowinski 2020) and for statistical analysis in populations (Sim et al. 2009), aggregation of image and clinical brain data (Nowinski et al. 2014a), dealing with data explosion (Nowinski 2016), radiology reporting (Nowinski 2016), and brain knowledge communication (for both doctor-to-doctor and doctor-to-patient) (Nowinski 2016).

The abovementioned operations and tools are incorporated into the atlasing software platforms, mostly to enable and enhance the atlas use. However, there exist numerous stand-alone tools suitable for atlas creation and use that are not incorporated directly into the created brain atlas platforms, such as *FreeSurfer*, a suite of tools for a cortical surface generation and quantification of functional, connectional and structural properties of the human brain (Fischl 2012) extended recently with the probabilistic atlas of the thalamic nuclei (Iglesias et al. 2018); *SPM* for a neuroanatomical variability assessment (Ashburner 2009); *FSL*, a comprehensive library of analysis tools for functional, structural, and diffusion MRI brain imaging data (Jenkinson et al. 2012); the *Medical Imaging Interaction Toolkit* (*MITK*) integrating two other powerful toolkits, the *Visualization Toolkit* (*VTK*) and the *Insight Toolkit* (*ITK*) (Wolf et al. 2005); and the *Vascular Editor* to create and edit vascular and, generally, tubular-like such as cranial nerve networks (Marchenko et al. 2010). Our experience shows that the tools directly integrated with the atlas have proved their value allowing any new atlas modules to be created and edited within the already existing neural context (Nowinski et al. 2012a; b).

Recently, a new generation of methods, tools, repositories, and neurotechnologies is planned or already under development. For instance, intensive technology development and validation is outlined under the *BRAIN Initiative* (BRAIN Working Group 2014; Jorgenson et al. 2015). Numerous atlas-related tools are being developed within brain big projects. For instance, a common automated preprocessing framework has been developed within the *Human Connectome Project* to bring multiple magnetic resonance imaging modalities together across a large cohort of subjects (Glasser et al. 2013). The *Neuroinformatics Platform* within the *Human Brain Project* develops tools to facilitate data acquisition and annotation, assignment of the anatomical location to data, and assembly of and access to spatially indexed information (Bjerke et al. 2018). Other examples of such tools developed within the *Allen Brain Atlas*, *BigBrain*, and *FSL atlas*, among others, are given in (Amunts and Zilles 2015). The *Scalable Brain Atlas* is a collection of web services that provides unified access to a large collection of public brain atlasing resources for the human and non-human species (Bakker et al. 2015).

Therefore, despite relatively slow progress in the development of the brain atlas enabling functionality so far in comparison to that of the atlas content, the recent brain big projects will strongly drive this functionality development due to dramatic needs to handle big data including their storage, visualization, processing, analysis, and (most importantly) interpretation. Moreover, technology advancement will enhance the brain atlas functionality development to create new atlases, such as a holographic brain atlas (Petersen et al. 2019) (note that much earlier we proposed to use holography in an atlas-enhanced operating room for the future (Benabid and Nowinski 2003)).

### Evolution of Atlas Availability

The human brain atlases reviewed above are available to the community in various ways and this availability can be considered in terms of what is available and how it can be accessed.

The atlases are available on two major media, print and electronic, resulting in three categories: print atlases, electronic atlases on various platforms, and transitional atlases from the print to the electronic medium.

The early maps and atlases were available in a print form. The transition from the print to electronic medium has been done via two channels by (1) creating both print and electronic (bi-media) atlases, and (2) derivation of early electronic atlases from print materials by direct atlas plate digitization with no content change or with content extension by postprocessing and enhancement. Tremendous developments in computing enable almost an unlimited growth of electronic brain atlases on numerous platforms ranging from mobile solutions to leading-edge supercomputers.

Electronic brain atlas platforms can generally be classified along multiple divisions: stand-alone versus web-based; stationary versus mobile; low-cost versus high-end workstations; with standard interaction and display versus VR-enhanced, augmented reality (AR)-enhanced and holographic display; standard computer versus supercomputer; and single computer versus computer clusters, networks, and cloud computing.

In neuroscience research, it is usually required to provide a brain atlas within a web-based solution. In general, brain atlases can run on various platforms. For instance, we have developed electronic brain atlases available and running on several platforms, including stand-alone plug-in library (Nowinski 2009); workstation (Nowinski 2009); notebook and desktop (for Windows and MAC) (Nowinski et al. 2005a; 2011b; Nowinski and Chua 2014); Internet-based (Nowinski et al. 2002a); mobile (iPhone (Nowinski et al. 2009c), iPad (Nowinski and Chua 2013b), Android (Nowinski et al. 2014c); and VR-enabled (Serra et al. 1997). It is worth mentioning that the latter pioneering 3D brain atlas, employing a VR environment with a 3D natural interaction and stereoscopic display, was completed as early as in 1997. Brain big projects, however, such as *The Human Brain Project*, require leading-edge supercomputers (Amunts et al. 2016).

From a user’s standpoint, we distinguish three levels of accessibility: non-accessible, private (with limited or unlimited access), and public (with registered or unregistered access; free or payable).

The non-accessible level means that the atlas is published by its creators and available only to them, and the community is aware of the atlas but has no access to view it completely nor use it. This is probably the most common situation. Private access indicates accessibility of the atlas to a certain group of users, such as members of a consortium. Public access implies that any user may have access to the atlas after meeting certain condition(s), such as registration and/or payment. For instance, the print atlases are public, payable with unregistered access. Most educational electronic brain atlases are public and payable, such as *Voxel-man* (Hoehne 2001), *The Human Brain in 1492 Pieces* (Nowinski et al. 2011b), *Focus Digital Anatomy Atlas. Neuroanatomy* running on iPhone and iPad (Focus Medica), and *Human Anatomy Atlas* (Visible Body n.d.). Our latest and most advanced atlas *The Human Brain, Head and Neck in 2953 Pieces* (Nowinski et al. 2015a) is public, free of charge with registration required by its publisher at http://www.thieme.com/nowinski/.

Although restricted access may constrict in some cases atlas availability, we may guess that overall this availability substantially grows over time, as the numbers of both atlas creators and their users have been rising tremendously. This guess is corroborated by Table 1 that provides the numbers of publications over time cited on PubMed under the term “human brain atlas” indicating the growth over 470 times from 1 publication in the year 1950 to 474 publication in the year 2018. Between years 2010–2018 this growth was almost 12 fold. The overall number of citations is 4350.

 The same term “human brain atlas” searched on Google Scholar gives “about 762,000 results”.

**Table 1:** Number of citations under the term “human brain atlas” on PubMed versus years of publications (as of 18 May 2020)

Year	Number	Year	Number
1950	1	2011	174
1960	1	2012	213
1970	3	2013	241
1980	5	2014	297
1985	18	2015	312
1990	16	2016	337
1995	22	2017	362
2000	42	2018	474
2005	121	2019	389
2010	185	2020	132

## Generations of Human Brain Atlases

Observing the evolution of human brain maps and atlases, four atlas generations can be distinguished, namely: (1) early cortical maps, (2) print stereotactic atlases, (3) early digital atlases, and (4) advanced brain atlas platforms. From a time-frame standpoint, approximately the first atlas generation was developed in the first half of the 20th century (with the major maps published in the first three decades), the second generation in the second half of the 20th century (with the majority of atlases published in its first four decades), the third generation in the *Decade of the Brain* (and a handful of atlases a little earlier), and the fourth generation in the 21st century, the century of the brain and mind.

**Fig. 1:**
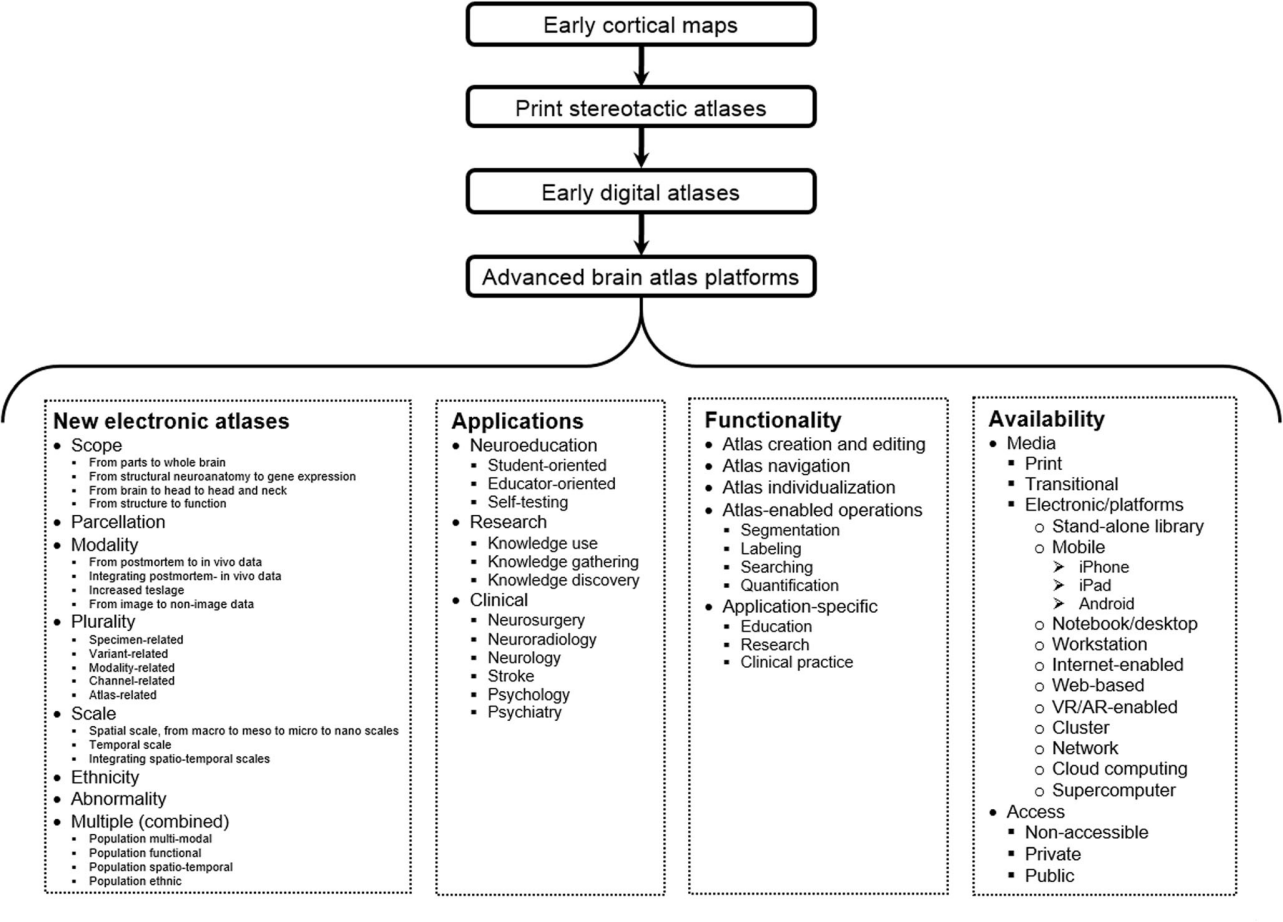
A human brain atlas evolution diagram with four generations and four categories: content (only for the new electronic atlases), applications, functionality, and availability, each subsequently divided into sub-categories

The above review, generations of atlases, and their present state are summarized in a form of the human brain atlas evolution diagram in Fig. 1.

## Discussion

The human brain atlases have been evolved tremendously, especially in recent decades, in multiple directions, as captured diagrammatically in Fig. 1. This evolution has been driven by sophisticated imaging techniques, advanced brain mapping methods, vast resources of brain data accumulated at an unprecedented rate, analytical strategies, and powerful computing. The effects of this explosive growth span from a few hand-drawn maps to multi-atlases, from print editions to web-based repositories, from 2D to nD, from determinist to probabilistic, from unimodal to multi-modal, from a cortical organization to an all-level brain organization from genes to the whole brain, from normal to pathologic, from gross to nanoscales, and various combinations of these above, among others. Several papers and reports have addressed the future trends that can be expected in the human brain atlas evolution, mainly in terms of an atlas content (Toga et al. 2006; Evans et al. 2012; Amunts et al. 2014; BRAIN Working Group 2014).

The brain atlases have been employed in a wide spectrum of applications and their usefulness depends not only on the atlas content, but also on functionality and availability. Hence this review has been conducted from these four perspectives: content, applications, functionality, and availability, in contrast to other works limited mostly to atlas content.

Content-wise, the human brain atlases have evolved from a few hand-drawn maps to an atlas as a collection of maps and images; to multi-atlases; to repositories of multi-modal brain images in health and disease; to heterogeneous databases; to composable, manipulable and explorable 3D and, generally, nD cerebral models; to platforms for brain knowledge aggregation and integration; to brain atlas data at macro, meso, micro, nano, and hybrid scales with the resolution ranging from the whole brain to synapses; and to large databases with massive amounts of data aiming to discover knowledge being developed within multi-center and/or multi-national projects and initiatives.

In an over century-long process of the human brain map and atlas creation, we have distinguished four generations: (1) early cortical maps (created in the first half of the 20th century), (2) print stereotactic atlases (published in the second half of the 20th century), (3) early digital atlases (produced predominantly in the *Decade of the Brain*), and (4) advanced brain atlas platforms (being developed in this century). We also noticed that every two decades mark major progress in the human brain atlas evolution. The first stereotactic brain atlases were created in the 1950th, the first digitized brain atlas was developed in the 1970th, the introduction of electronic brain atlases to clinical practice began in the 1990th followed by an explosion in brain atlas development propelled by the brain big projects that started in the 2010th .

The development of electronic brain atlases spans the last two generations and in this area we have distinguished five avenues. The recent and most prominent avenue is the creation of new electronic brain atlases. We give numerous examples of vast activities in the area of atlas content development heading in at least 23 various (though non-exhaustive) directions, which are categorized in eight groups taking into account scope (content extent), parcellation, modality, plurality, scale, ethnicity, abnormality, and a mixture of them.

Application-wise, brain atlases are employed in education, research, and clinical practice. The role and usefulness of the brain atlases have been expanding both within the research area and beyond it. The main atlas application area is research and brain knowledge gathering ranging from knowledge capturing to knowledge aggregation to knowledge discovery. Other areas of atlas applications include human brain mapping (spanning research and clinical practice), stereotactic and functional neurosurgery, neuroeducation, and specific areas, such as neuroradiology, neurology, psychology, stroke, and psychiatry.

The major application-wise shift has been from research to clinical practice, particularly in stereotactic and functional neurosurgery to treat patients. A brain atlas importance and potential in clinical applications have been raised and addressed by a few authors (Mori et al. 2013; BRAIN Working Group 2014). In fact, the development of brain atlas-based clinical applications for prediction, diagnosis, and treatment has been a major focus of our work. Neurosurgery planning and assessment (Nowinski 1998; 2001; 2009; Nowinski et al. 2010) was our first clinical application with anatomic, functional, and vascular atlases created. Our solutions in stereotactic and functional neurosurgery have been licensed to 13 surgical companies and integrated with surgical workstations of the leading companies, namely, Medtronic, Brainlab, and Elekta (Nowinski 2009). In addition, we have developed working prototypes in other fields for atlas-assisted brain pathology detection (Nowinski 2020), quantification of cerebral lesions (Nowinski et al. 2006), segmentation and labeling of pathological neuroimages with tumors causing a mass effect in brain cancer (Nowinski and Belov 2005), stroke management (Nowinski et al. 2006; Nowinski 2020), and stroke outcome prediction (Nowinski et al. 2014a). A vast, still unexploited, potential of brain atlas in neuroradiology has been addressed in (Nowinski 2016) describing nine applications for which working prototypes (proofs of concept) we developed earlier and presented at clinical meetings. However, despite several examples of brain atlas use in clinical applications (as products or working prototypes), these applications are still lagging behind the progress in the development of the atlas content. One of the main obstacles in introducing the brain atlas solutions to clinical practice is their validation, which is tedious, time-consuming, and costly; particularly, clinical validation is beyond a reach of a research lab because of its high cost.

In contrast to the content-wise atlas development being widely carried out by numerous groups as well as by national and multi-national consortia, the development of atlas functionality has been relatively neglected, until recently as the problem of managing data explosion requires powerful, suitable, and dedicated tools.

The early atlases evolved from bare, hand-drawn maps to print stereotactic atlases with images scalable using an overhead projector to electronic deformable atlas platforms to VR- and AR-enhanced atlases to atlas-engines meaning the atlases serving as tools by themselves that support brain data management, neuroimage processing and analysis, decision making, and knowledge discovery.

From an application standpoint, the tools providing an atlas with its supporting functionality belong to three categories: (1) educational tools to explore the atlas, test knowledge, and prepare teaching materials (that can be grouped as student-oriented, educator-oriented, self-testing, and a mixture of them); (2) research tools enabling brain investigation and knowledge discovery; and (3) clinical tools to allow the clinicians to better prevent, diagnose, treat, and cure brain diseases.

Efficient and user-friendly tools are, in particular, required, in education. We have attempted to develop new education tools going beyond those available in standard educational atlases, such as *Voxel-man* (Hoehne 2001) or *Interactive Head & Neck* (Berkovitz et al. 2003). These tools enable novel educational use of the atlas, such as self-testing and classroom assessment (Nowinski et al. 2009c) available on notebooks and mobile devices, interdisciplinary education across neuroanatomy-neuroradiology-neurology (Nowinski et Chua 2013a), advanced education for residents and clinicians with a user’s “de/composable” content and context (Nowinski et al. 2014b; 2015a), and patients’ education and instruction (Nowinski 2016).

From a usage standpoint, atlas tools can be classified into two broad categories: general and specific. General tools support typical atlas-enabled operations, such as segmentation, labeling, manipulation, quantification, and querying. Specific operations are those customized to a certain field and/or particular use, such as automatic testing, generation of teaching materials, ROIs/VOIs generation and analysis, targeting, safety analysis, postoperative assessment, locus-driven analysis, decision making support, prediction of occurrence and outcomes, scan interpretation, knowledge communication, and a combination of them. From an integration standpoint, atlas-related tools can be stand-alone or directly integrated with the atlas platform.

Availability-wise, the major developmental step was obviously from print to digital atlases. Enormous progress in computing enables almost unlimited development of digital brain atlases to run on numerous platforms ranging from mobile solutions to notebooks to interactive web-based visualization platforms to VR/AR systems to high-end workstations to computer clusters, networks, and leading-edge supercomputers.

The atlas availability substantially grows over time with the numbers of both atlas creators and their users tremendously raising. If approximated by the growth of the human brain atlas publications on PubMed, the atlas availability growth from the year 1950 to the year 2018 would be over 470 times.

This work has several limitations. We have tried our best to make this state-of-the-art review in the human brain atlas evolution as complete as possible. However, the overall number of publications about this subject on PubMed is vast of 4350 (and about 762,000 references on Google Scholar) and rapidly growing, which makes a fairly complete state-of-the-art review quite difficult (if possible at all). In some areas, such as clinical applications, any relevant research publications may simply not exist, and the names of atlas creators and developers may not be disclosed by the providers to the atlas users (as, for instance, is in the case of our brain atlases licensed to surgical companies).

The brain atlas content evolution is divided at two levels into 8 groups at the first and 23 directions at the second level. We believe that the categorization of the atlas content development into these 8 groups covers the whole landscape, though it is not unique and other criteria might be applied. This categorization is neither distinctive and some groups may overlap, for instance, the increasing scale may result in the increasing scope. The overall 23 directions in the atlas content development are not exhaustive and could be finer, especially in the last (combined) group. Likewise, the ethnicity and abnormality groups could be subdivided into directions, each for a specific ethnicity or disease, respectively.

The approximation of the atlas availability growth through the number of publications about human brain atlas may be underestimated even by a few orders. Usually a publication about a free atlas attracts a plethora of its users, and even the number of citations may not be representative. For instance, our free brain atlas (Nowinski 2017b) has a download-to-citation ratio of 160.

This review is restricted to the human brain atlases. Several authors have overviewed non-human brain atlases for various species, including primates (marmoset, mouse lemur, squirrel monkey, macaque, and chimpanzee by Thiebaut de Schotten et al. (2018)), rodents and marsupials (rat, mouse, and opossum by Bakker et al. (2015)), and other animals by Hess et al. (2018). It is also worth mentioning that several human spinal cord atlases have been constructed, for instance, by Taso et al. (2013) and Lévy et al. (2015).

Finally, this review reflects a personal perspective and three-decade-long experience of the author in the field with 35 diverse human brain atlases created, where 15 of them have been released for the global use by Thieme Medical Publishers.

Making an overview of a field also encourages an attempt to predict future developments in brain atlasing. On one hand, the future brain research directions are well determined in the brain big projects, such as the BRAIN Initiative that sets six grand goals (BRAIN Working Group 2014). These efforts will result in the acquisition of more and more massive amounts of data and the creation of more advanced and complex brain atlases with an ever-growing scope, population, and spatial and temporal resolutions, additionally empowered by more advanced tools. On the other hand these efforts keep increasing a sort of atlas landscape inhomogeneity as well as difficulty in the atlas standardization and the integration and interpretation of various outcomes. Moreover, as the majority of efforts is devoted to brain atlas-related research, we can expect a growing imbalance and chasms among research, clinical, and educational applications of human brain atlases.

There are at least three central components related to atlas standardization, namely, an atlas coordinate system, a core reference cerebral model, and a brain atlas platform architecture.

The two most widely used coordinate systems in the neuroscience community are the Talairach system (Talairach and Tournoux 1988) and the Montreal Neurological Institute (MNI) system, and any coordinates of the latter can be converted to the Talairach space (Chau and McIntosh 2005). The Talairach coordinate system has become the standard reference for reporting the brain locations in scientific publications, though its definition is not unique. The Talairach system is based on the anterior (AC) and posterior (PC) commissure line and its center is located at the AC point landmark. However, the AC and PC point landmarks can be defined at least in four different ways resulting in a substantial discrepancy among the coordinates depending on a selected landmark definition (Nowinski 2001b). Typically the centers of the AC and PC structures are taken as the point landmarks, while the originally defined point landmarks by Talairach are beyond the AC and PC structures (consequently, for instance, the AC is missing on the coronal plane passing through the center of the coordinate system (and in my print version of his atlas, prof. Talairach “corrected” that by manually drawing it)).

A core reference high-quality cerebral model is missing in neuroinformatics. An example of such a long-lasting reference model for the cerebral cortex are Brodmann’s areas (Brodmann 1909). Brodmann’s areas, though being one century old and based on a single brain specimen, are most widely used and remain until today applicable references in human brain mapping to correlate functional activations to the underlying neuroanatomy (Amunts and Zilles 2015), despite the creation of more advanced and accurate cortical maps (Glasser et al. 2016). For a certain period the Talairach and Tournoux (1988) atlas has played a similar role for the whole brain, despite its well-known limitations including spatial consistency as quantified by Nowinski and Thirunavuukarasuu (2009). Another example is the Schaltenbrand and Wahren (1977) atlas that for a few decades until the present remains the reference in stereotactic and functional neurosurgery.

**Fig. 2:**
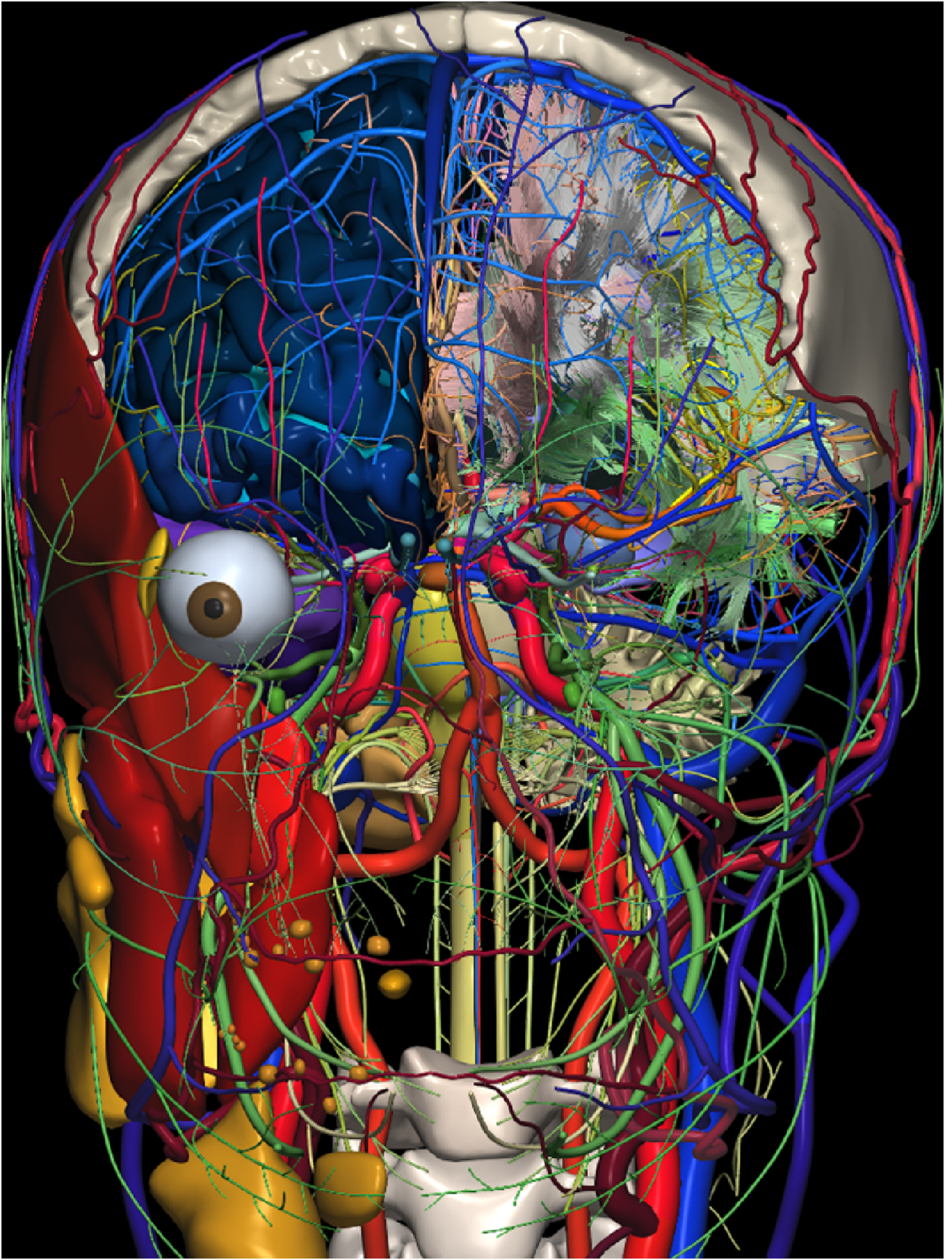
The virtual decomposable brain model extended to the head and neck with about 3,000 fully segmented, labeled, and color-coded 3D components. Shown here: the right central nervous system with the cerebrum (parcellated into gyri and sulci), cerebellum, brainstem, and cervical spine; deep gray nuclei; cerebral ventricles; white matter (deep and posterior fossa); white matter tracts; right visual system; auditory system; intracranial arteries; intracranial veins; dural sinuses; cranial nerves with nuclei (partly exposed on the left side); right head (masticatory) muscles; right glands; upper skull with the frontal bone removed; cervical spine (3rd and 4th cervical vertebrae); extracranial arteries; and extracranial veins

The construction of the core virtual brain model of the highest possible quality is a complicated, tedious, and time-consuming process, which requires sophisticated, dedicated, and precise tools and, of course, the state-of-the-art data, besides meticulous attention to details. Therefore, such a virtual brain model shall be built incrementally. We have attempted to create this kind of virtual brain model from multi-modal, multi-sequence scans of a living specimen (in a process which took almost 15 years until funding lasted), see Fig. 2. This model has been designed as modular (Nowinski 2017b) with its consecutive modules being developed and validated (including cortical areas and subcortical structures (Nowinski et al. 2012a), white matter tracts (Nowinski et al. 2012b), intracranial vasculature (Nowinski et al. 2011a), cranial nerves and nuclei (2012c), head muscle and glands (2013c), extracranial vasculature (2015b), skull (2015c), and systems, while releasing subsequent five versions (termed *The Human Brain in 1492/1969/2953 Pieces*) for public use (Nowinski et al. 2011b; 2015a; Nowinski et Chua 2014). Our effort, though uncompleted, has demonstrated the feasibility of this approach.

Although the advantages of population atlases are enormous and obvious, we believe that these atlases shall be constructed around a very detailed, accurate, fully segmented, completely labeled, validated, and deterministic core model of a virtual brain created with the highest quality possible and accepted as the reference standard (similarly as Brodmann created his long-lasting standard for the cortical areas from a single specimen). Moreover, the use of a single specimen enables its continuous rescanning to create new modules with advances in imaging technology (for instance, to build our virtual brain model, the same specimen was rescanned for over 10 years on various 1.5T, 3T, and 7T as well as CT and US (ultrasonography) scanners). Creating a population brain atlas even with a high number of specimens but without ensuring the highest quality and thorough validation just increases the abovementioned inhomogeneity of the field with a difficulty to cross-relate various atlases.

The final factor that might potentially counterbalance this atlas inhomogeneity trend is the establishing a standardized, general architecture of the human brain atlas platform supporting equally research, clinical, and educational applications and enabling the clinicians to grow the initial core brain model with their own new data. We believe that the future human brain atlas-related research and development activities shall be founded on and benefitted from such a standard framework containing the core virtual brain model cum the brain atlas platform general architecture.

## Summary

We have attempted here to track enormous transformational advances in the human brain atlas evolution from hand-drawn cortical maps to print brain atlases to digital atlases with tools to multi-modal and population atlases in health and disease to mega multi-atlases across the lifespan to atlas platforms at macro, meso, micro, and nanoscales, as diagrammatically summarized in Fig. 1. This atlas evolution review differentiates from other works, usually focusing on the atlas content mostly in research applications, as we take here a wider perspective and analyze this evolution in four categories: content, applications, functionality, and availability. Four generations of human brain atlases are distinguished, namely, early cortical maps, print stereotactic atlases, early digital atlases, and advanced brain atlas platforms. The development of electronic brain atlases spans the last two generations and in this area we identify five avenues, the recent and most prominent is the creation of new electronic brain atlases. The brain atlas content evolution in this avenue is categorized in eight groups taking into account scope, parcellation, modality, plurality, scale, ethnicity, abnormality, and a mixture of them, in which, in turn, the atlas developments are heading in 23 various directions.

We suggest that the future human brain atlas-related research and development activities shall be founded on and benefitted from a standard framework containing the core virtual brain model cum the brain atlas platform general architecture.

## Footnotes

1 Note that we were the only research group that received rights from the publisher to use these atlases.
